# Self-Defining Memories and Well-Being in European American and Chinese Emerging Adults

**DOI:** 10.3390/bs16030408

**Published:** 2026-03-11

**Authors:** Wanzi Li, Çağla Duman, Wenxuan Hou, Jessie Bee Kim Koh, Qi Wang

**Affiliations:** 1 Department of Human Development and Psychology, Cornell University, Ithaca, NY 14853, USA; wl774@cornell.edu (W.L.); cd626@cornell.edu (Ç.D.); wh493@cornell.edu (W.H.); 2 Department of Applied Psychology, Chinese University of Hong Kong, Shenzhen 518172, China; bk94@cornell.edu

**Keywords:** self-defining memory, well-being, meaning integration, emotion intensity, emerging adults, culture

## Abstract

This study examined self-defining memories (SDMs) in relation to well-being in European American and Chinese emerging adults. European American (*N* = 118) and Chinese participants (*N* = 122) each generated two SDMs; rated the emotionality, importance, and clarity of the memories; and completed well-being measures. Memory narratives were coded for meaning integration, affect, specificity, and content. Chinese participants made more references to others in their narratives than did European Americans, who recalled more specific events. European American participants reported both higher depression and higher flourishing than Chinese participants. Furthermore, while meaning integration, positive emotion words, memory clarity, and achievement content were related to psychological well-being in European Americans, positive emotion words and personal importance were related to well-being in Chinese. Regressions further showed that positive emotional intensity of SDMs was associated with greater positive affect states only among Chinese, whereas meaning integration was associated with reduced negative affect states only among European Americans. These original findings highlight the important role of autobiographical memory in well-being in specific cultural contexts.

## 1. Introduction

Autobiographical memory serves to support a coherent sense of self, facilitate social connections, and guide future behavior (Fivush, 2011; D. B. Pillemer, 1998; Q. Wang, 2013). A substantial body of work suggests that the way people remember, interpret, and experience their memories is associated with their identity and well-being (Conway & Pleydell-Pearce, 2000; Garcia, 2014; Gehrt et al., 2023; Rathbone et al., 2015). In particular, self-defining memories (SDMs) are vivid, emotionally charged, and frequently recalled autobiographical memories that are closely related to personal values and self-identity (J. A. Singer & Salovey, 1993). Prior work has revealed close relations between characteristics of SDMs and psychological well-being (Adler et al., 2016; Blagov & Singer, 2004; McKay et al., 2024; J. A. Singer et al., 2013). Critically, autobiographical memories and their psychosocial functions are shaped by cultural meaning systems (Duman & Wang, 2025; Q. Wang & Suo, 2023; Q. Wang et al., 2018). Yet no study to date has examined the link between characteristics of SDMs and psychological well-being in different cultural contexts. The present study addresses this gap by investigating SDMs in relation to psychological well-being among European American and Chinese emerging adults.

### 1.1. Characteristics of Self-Defining Memories

Autobiographical remembering involves the integration of perspectives, interpretations, and evaluations of significant personal experiences to craft a personal history that serves immediate and long-term goals (Conway et al., 2004; Fivush, 2011; McAdams, 2001; Q. Wang, 2013). Through autobiographical remembering, individuals structure the past into narratives, derive purpose and meaning, and construct a sense of personal identity (McAdams & McLean, 2013). As a special form of autobiographical memory, self-defining memories (SDMs) are vivid, emotionally intense, and highly self-relevant, often centering on personal concerns and conflicts (J. A. Singer & Salovey, 1993). It is proposed that these memories play a special role in supporting a coherent sense of self by allowing individuals to make meaning from important life experiences (Blagov & Singer, 2004; J. A. Singer & Salovey, 1993; J. A. Singer et al., 2013). Research has further shown that SDM narratives often emerge to include dimensions of meaning integration, affect, specificity, and content (Blagov et al., 2022; Blagov & Singer, 2004).

Meaning integration involves deriving personal significance from remembered events and further constructing narratives that articulate lessons about the self, others, or the world (Blagov & Singer, 2004). Integrated narratives often contain explicit meaning-making, in which individuals take an evaluative stance toward the experience and consider how the event fits within the broader themes of their life stories (J. A. Singer & Blagov, 2000, 2004). Through this process, specific episodes may become linked to one’s identity and personhood, being integrated into a coherent narrative understanding of the self (Fivush, 2011; McAdams & McLean, 2013). Research suggests that meaning integration in SDMs varies across individuals and event-related characteristics, with more extensive meaning-making in people with higher self-esteem and for events with greater subjective impact (Liao et al., 2018; Wood & Conway, 2006).

Affect, including both emotional valence and intensity, is another commonly studied dimension of SDMs (J. A. Singer, 1990; Moffitt & Singer, 1994). Emotional valence refers to the positive or negative tone of a memory, whereas emotional intensity pertains to the strength or magnitude of the associated emotional response (McLean & Lilgendahl, 2008). Studies have shown that the affective quality of SDMs is related to goal relevance and perceived personal importance of the remembered event, with successful goal attainment and greater importance typically associated with more positive emotions (Moffitt & Singer, 1994; Ritchie et al., 2014). Affect may also shape how people process and interpret their experiences, and some work suggests that memories with a more negative emotional tone can elicit greater meaning-making as individuals reappraise difficult experiences through narrative processing (McLean & Pratt, 2006; Pals & McAdams, 2004; Thorne et al., 2004).

In addition to meaning integration and affect, SDMs also vary in their narrative structure and in the degree of sensory, spatial, and temporal details in memory narratives (J. A. Singer & Blagov, 2000). Specific SDMs refer to unique, one-time events that occurred at a particular time and place, typically lasting less than a day and containing concrete details about what happened and how one thought or felt. In contrast, non-specific, general SDMs involve repeated or extended experiences that unfolded across multiple occasions or over extended periods, and they tend to contain more abstract summaries of the past rather than discrete episodic details (Blagov & Singer, 2004; D. B. Pillemer 1998). It has been shown that constructing specific memories can support important functions in daily life, including guiding behavior, informing identity, and fostering social connections (Guan & Wang, 2022; D. Pillemer, 2003; D. Pillemer et al., 2003).

Finally, SDMs also differ in the central concern emphasized within the narrative (Thorne & McLean, 2001). The thematic content of SDMs may reflect individuals’ enduring concerns and motivational orientations (Blagov et al., 2022; Moffitt & Singer, 1994). They may further vary across periods of life, reflecting the types of experiences individuals undergo (Enz & Talarico, 2016; McLean & Thorne, 2003; J. Singer et al., 2007). Previous research has identified several common themes in SDMs, including safety, exploration, interpersonal relationships, achievement, and guilt or shame (Thorne & McLean, 2001). Importantly, given that cultural meaning systems critically shape the self and memory processes (Q. Wang, 2013, 2021), it is essential to examine the construction of SDMs and their narrative features in different cultural contexts.

### 1.2. Cultural Construction of Autobiographical Memory

Culture shapes how the self is construed and how personal experiences are remembered and structured into narratives (Ross & Wang, 2010; Q. Wang et al., 2017). In Western, particularly European American, cultures, a greater emphasis is placed on an independent self-construal that is autonomous and unique, whereas in East Asian cultures, such as China, a greater emphasis is placed on an interdependent self-construal that is grounded in connectedness and modesty (Heine & Hamamura, 2007; Markus & Kitayama, 1991; Q. Wang, 2016). Cultural values pertaining to the self may condition the process of personal storytelling, where individuals draw on culturally salient metaphors, plots, and narrative structures to craft memory narratives that align with culturally appropriate ways of understanding the self and the past (McAdams, 2013; Q. Wang et al., 2017). Together, variations in self-construal and storytelling practices influence the construction, meaning-making, and narrative organization of autobiographical memories across cultural contexts (Q. Wang, 2013, 2016).

Specifically, cultural differences have been observed in meaning integration, affect, specificity, and content of autobiographical memories. Research has shown that European Americans often engage in meaning-making by constructing redemptive narratives, in which past difficulties are reframed as experiences of overcoming struggles and achieving growth and positive transformation (McAdams, 2006a, 2006b). In contrast, memory narratives by East Asians frequently revolve around themes of morality, effort, and intellectual advancement, emphasizing the extraction of lessons from the past to reinforce culturally shared ideals (Duman & Wang, 2025; Q. Wang et al., 2024; Q. Wang & Conway, 2004). Whereas memory narratives provided by European Americans tend to be specific and emotionally expressive, highlighting the rememberer’s unique personal history and subjective perspective, East Asian narratives tend to be more general and less emotionally elaborate, in line with Asian cultural norms of modesty and emotional restraint for maintaining social harmony (Q. Wang, 2001, 2006; Q. Wang & Conway, 2004; Q. Wang & Yang, 2023). Relatedly, European Americans often recall self-focused events and construct narratives centered on personal achievement and growth, whereas East Asians tend to remember socially oriented memories that highlight interactions and relationships (Conway et al., 2005; Jiang et al., 2023; Kulkofsky et al., 2010; Q. Wang, 2001, 2006; Q. Wang & Conway, 2004).

Although research on culture and autobiographical memory has revealed important insights, empirical work directly examining SDMs across cultural contexts remains limited. Jobson and O’Kearney (2008) observed that the characteristics of SDMs varied by culturally emphasized domains, such that individuals from Western cultures provided more elaborate autonomy-related memories than relationship-related ones, whereas those from Asian cultures showed the opposite pattern. Y. Wang and Singer (2021) found that compared with American college students, Chinese students remembered SDMs with more positive affect and greater perceived importance, although their memories also revolved more around guilt and shame themes, particularly in academic contexts where self-improvement motivations tend to be salient. These findings suggest that the construction and experience of SDMs may manifest variably in different cultural contexts, which may be further reflected in the relation of SDMs to well-being.

### 1.3. Memory and Well-Being in Cultural Context

The characteristics of SDMs, including meaning integration (Adler et al., 2016), emotional valence and intensity (McKay et al., 2024), specificity (J. A. Singer et al., 2013), and thematic content (Blagov & Singer, 2004), have been linked to psychological well-being. Among these characteristics, meaning integration has received particular attention, given its central role in identity construction and adaptive functioning (Gillies & Neimeyer, 2006; Park, 2010). Specifically, meaning-making may function as an emotion regulation mechanism, whereby finding silver linings in negative experiences and reinterpreting past events in a more positive light may buffer against stress and other negative feelings (Cox & McAdams, 2014; Gu et al., 2025; Tavernier & Willoughby, 2012). Indeed, research has found that meaning-making is associated with low distress and low negative affect in recall (Gu et al., 2025; Korte et al., 2012; Meisels & Grysman, 2021).

Affective qualities of SDMs have also been linked to well-being, whereby self-reported negative emotions tend to be associated with distress and positive emotions are often related to life satisfaction and psychological adjustment (Blagov et al., 2022; Lilgendahl & McAdams, 2011; Siedlecki et al., 2015; J. A. Singer & Blagov, 2004). Moreover, memory specificity plays an important role, whereby reduced specificity has been linked to psychological disorders, such as depression, bipolar disorder, and PTSD (McKay et al., 2024; J. M. G. Williams et al., 2007; Wright et al., 2022). Finally, memory content focus is also related to psychological functioning, such that themes of disrupted relationships and threat were associated with greater psychological distress (Blagov & Singer, 2004).

Importantly, the strength and direction of these associations may vary across cultural contexts. For example, affective changes in negative parent–child relationship memories were associated with psychological well-being among Asians but not European Americans (Song & Wang, 2025). Whereas recalling detailed memories was linked to more adaptive coping in European Americans, it was associated with elevated depressive symptoms, lower adaptive skills, and greater negative affect in East Asians (Q. Wang et al., 2018). During the COVID-19 pandemic, Asian Americans recalled more positive memories related to social distancing than did European Americans, despite reporting memories with broadly similar content, and only among Asian Americans were more positive social-distancing memories related to better psychological well-being (Q. Wang & Suo, 2023). These findings suggest that the associations between memory characteristics and well-being may sometimes differ across cultural contexts. They are consistent with the person–culture fit framework positing that autobiographical memories in alignment with culturally prioritized self-goals and meaning systems are likely to be associated with positive psychological outcomes (Jobson, 2011; Reese et al., 2017; Suo & Wang, 2022; Q. Wang et al., 2018).

Notably, well-being is generally conceptualized as a multifaceted construct encompassing affective and cognitive evaluations of one’s life (Kahneman et al., 1999; Diener et al., 2003). Many theorists further distinguish hedonic components from the broader indicators of positive functioning, such as meaning, purpose, social integration, and flourishing, when describing overall mental health (Diener et al., 2009; Ryff & Singer, 2008; Schimmack, 2008). Accordingly, well-being is often operationalized in terms of a combination of features with emotional and psychological indicators (Keyes, 2002). At the same time, operationalizations vary across studies, and previous research linking autobiographical memory to well-being has often focused on indicators of affective experience and distress (e.g., depression; Sumner et al., 2010), along with proximal processes, such as emotion regulation and coping (e.g., Hermans et al., 2005). It remains unclear whether the patterns identified in the literature would extend to SDMs in different cultural contexts.

### 1.4. The Present Study

The present study examined self-defining memories and their relation to psychological well-being in a cross-cultural context. European American and Chinese emerging adults remembered and narrated two SDMs and rated each memory on emotional intensity, clarity, and personal importance. Their memory narratives were coded for meaning integration, affect, specificity, and content. Participants also completed measures of psychological well-being, including flourishing experiences, depressive symptoms, and positive- and negative affect states. While the influence of culture on psychological processes or constructs can be studied at multiple levels (Q. Wang, 2018, 2026), we adopted in the current study a group-level approach, examining the cultural construction of SDMs in European American and Chinese participants, as well as an individual-level approach, examining the relation of SDMs to psychological well-being across individuals. Furthermore, following prior work (Diener et al., 2010; Westerhof & Keyes, 2010), we operationalized well-being using complementary indicators of positive functioning and maladjustment to capture affect (i.e., positive and negative affect states), psychological strength (i.e., flourishing), and distress (i.e., depressive symptoms). We examined these outcomes separately and as a composite index for overall well-being.

In line with prior research, we predicted that compared with Chinese participants, European Americans would provide SDMs with greater meaning integration, more emotional expressiveness, greater specificity, and more self-focused content. We further expected the associations between SDM characteristics and well-being to show similarities and differences across cultures. Specifically, in line with the person–culture fit framework (Chen, 2018; Q. Wang et al., 2018), we expected that, among European Americans, meaning integration, specificity, positive emotions, and achievement themes would show positive associations with flourishing, positive affect states, and overall well-being, and negative associations with depressive symptoms and negative affect states. Among Chinese participants, we expected that positive emotions, social orientation, and relationship themes would show positive associations with flourishing, positive affect states, and overall well-being, and negative associations with depressive symptoms and negative affect states.

In addition to culture, gender may also play a role in the narrative construction of SDMs as they relate to well-being. Thus, we additionally examined whether self-reported gender was associated with variations in SDM characteristics and their links to well-being. Prior research suggests that women’s autobiographical narratives tend to be longer, more emotionally elaborate, and more focused on relational and communal themes than men’s narratives (Bauer et al., 2003; Bohanek & Fivush, 2010; Bohn & Berntsen, 2008), although gender differences often vary by context and methodology (Grysman & Hudson, 2013; Grysman & Wang, 2021). Guided by this literature, we hypothesized that women would produce SDMs that were more emotionally expressive and more socially focused than those of men.

## 2. Method

### 2.1. Participants

An a priori power analysis was conducted using G*Power 3.1 (Faul et al., 2009). For a 2 (Culture: European American vs. Chinese) × 2 (Gender: men vs. women) between-subjects design, a sample size of 210 was required to achieve a power of 0.95 to detect effects with a size of *f* = 0.25 at *α* = 0.05. Considering potential attritions, we recruited a larger sample of 244 young adults, including 120 from the U.S. and 124 from China. The U.S. participants were undergraduate students at Cornell University, all European Americans, and the Chinese participants were undergraduate students at the Chinese University of Hong Kong, Shenzhen. Four participants (*N*_US_ = 2 and *N*_CH_ = 2) were excluded for not following the instructions (i.e., not providing memory narratives). The final sample thus consisted of 240 participants, including 118 European Americans (76 women and 42 men, *M_age_* = 20.48, *SD_age_* = 1.24) and 122 Chinese (80 women and 42 men, *M_age_* = 19.70, *SD_age_* = 0.89). The research protocol was reviewed and approved by the Cornell University Institutional Review Board (IRB). Participants provided informed consent and received extra course credits for their participation.

### 2.2. Procedure

Participants took part in the study through an online survey. They first completed an SDM task and then rated the phenomenological qualities of their memories. Next, they completed three well-being scales and provided demographic information. Participants reported gender using a single demographic item with binary response options (i.e., male, female). The survey was developed in English and Chinese versions, and a translation–back-translation procedure was used to ensure meaning equivalence between the two versions.

### 2.3. Tasks and Measures

#### 2.3.1. Self-Defining Memory Task

A self-defining memory task was used following prior research (Blagov & Singer, 2004). Participants each recalled two SDMs according to a standard instruction in which they were told that “self-defining memories are vivid, emotionally intense, recalled repetitively, important to you and linked thematically to similar memories.” Participants were asked to recall and describe in detail two self-defining memories that were important to an enduring theme, issue or conflict in their lives. There was no time restriction for participants to complete the memory task.

After finishing describing the memories, participants provided their age at the time of each event. They then rated the phenomenological qualities of the memories. They first rated on the intensity of the emotions associated with each memory on a 7-point Likert scale (0 = not at all to 6 = extremely). Ratings were provided for 12 discrete emotions, including happiness, sadness, anger, fear, surprise, shame, disgust, guilt, interest, embarrassment, contempt, and pride. Mean ratings for positive and negative emotions were calculated, respectively, and averaged across the two memories for later analyses.^1^ Positive emotion rating scores had acceptable (*α* = 0.68) and good (*α* = 0.74) internal consistency in the U.S. and Chinese samples, respectively, and negative emotion rating scores had good internal consistency in both samples (*α*s > 0.80). Participants then rated the extent to which the memory was detailed and clear (0 = very vague to 6 = most vivid) and how personally important it was (0 = not at all to 6 = most important). The ratings were averaged across the two memories for analysis.

#### 2.3.2. Well-Being Scales

*Flourishing*. The 8-item Flourishing Scale measures self-perceived success in relationships, self-esteem, purpose, and optimism (e.g., “I am engaged and interested in my daily activities;” (Diener et al., 2010)). Participants were asked to rate each item on a 7-point Likert scale ranging from 1 (strongly disagree) to 7 (strongly agree). Participants’ ratings were then summed to obtain a total score. The scale showed good internal consistency in both samples (*α*s > 0.89).

*Depression*. A 24-item depression scale developed by Chang and Koh (2012) was used. Participants rated how well each item (e.g., “I feel hopeless”) described their experiences over the past 2 weeks using a 4-point Likert scale ranging from 1 (very rarely or never) to 4 (very often or always). Four items were reverse-scored, and item scores were summed to compute a total depression score. The measure showed good internal consistency (*α* = 0.88 for both samples).

*Affect states*. The Scale of Positive and Negative Experience (SPANE; Diener et al. (2010)) includes 12 items assessing how frequently participants experienced various positive (6) and negative (6) emotions over the past 4 weeks. Participants rated each emotion on a 5-point Likert scale ranging from 1 (very rarely or never) to 5 (very often or always). Positive and negative affect state scores were calculated by summing the respective ratings. The SPANE-Positive subscale demonstrated good internal consistency in both samples (*α*s > 0.87) as did the SPANE-Negative subscale (*α*s > 0.80).

### 2.4. Coding

For each memory narrative, three dimensions, including meaning integration, affect/emotionality, and specificity, were coded according to the SDM scoring manual developed by J. A. Singer and Blagov (2000). The content dimension in terms of the event theme of SDMs was coded according to the manual by Thorne and McLean (2001). In addition, the content dimension in terms of self- or social focus as well as memory length were coded following prior autobiographical memory research (Q. Wang, 2001; Q. Wang & Conway, 2004). Two trained coders who were blind to the hypotheses independently coded 20% of the data for interrater reliability estimates, with Cohen’s kappa and ICC > 0.84 for all variables. Discrepancies were resolved through discussion among the coders who then completed the coding of the remaining data. Each memory was coded for the following characteristics:

*Memory Length*: The number of words in each narrative (without punctuation marks) was counted to measure the length of memory narratives, as a potential covariate.

*Meaning Integration***:** Each memory was coded as either integrative or non-integrative based on whether the narrative conveyed a sense of meaning or a life lesson to the individual (J. A. Singer & Blagov, 2000). SDMs with meaning integration were scored as 1 and those without were scored as 0.

*Emotionality:* The number of times participants explicitly mentioned emotions in each narrative was counted to index emotional expressiveness. Positive emotion words (e.g., “happy,” “excited”) and negative emotion words (e.g., “sad,” “angry”) were counted separately, and emotion-expressive behaviors (e.g., “smiled,” “laughed”) were also included (Reese et al., 2017). Positive emotion words were summed to create a positive emotionality score, and negative emotion words were summed to create a negative emotionality score. Non-emotion words, even with positive or negative connotation (e.g., “success,” “adversity”), were not counted.

*Specificity:* Memories were coded as either specific or non-specific. A specific memory referred to an event that occurred on a particular date or within a time frame of less than one day, while a non-specific memory referred to general or repeated experiences. Specific memories were scored as 1 and non-specific memories were scored as 0.

*Event Type:* Memories were categorized based on the main theme of each narrative (Thorne & McLean, 2001). The categories were exclusive and included relationships, life-threatening events (LTE), recreation or exploration, achievement or mastery, guilt or shame, drug/alcohol/tobacco use, and unclassifiable events. Only memories focusing on relationships, LTE, and achievement were later analyzed; the other types of events were rare and not examined further.

*Other/Self Ratio:* The number of self-references (e.g., “I,” “me,” “myself”) and other-references (e.g., “he,” “she,” “my friend”) in each narrative was counted. Words such as “we” were counted twice, once as a self-reference and once as an other-reference. An other/self ratio score was then computed for each memory to index the social orientation of the memory content (Q. Wang, 2001; Q. Wang & Conway, 2004).

## 3. Results

### 3.1. Self-Defining Memory Narratives

Given that each participant reported two SDMs, continuous ratings were averaged across the two narratives, and categorical codes (i.e., meaning integration, specificity) were converted into frequency scores of 0 to 2, which were then submitted to analysis. Chinese participants (*M* = 310.93, *SD* = 227.03) provided significantly lengthier narratives than did European Americans (*M* = 89.02, *SD* = 95.06), *t*(163.30) = 9.93, *p* < 0.001. Note that narrative length can act as a confounding variable, given that longer narratives may inherently contain more information (e.g., emotion words, references to self and others, meaning integration), independent of the quality or content of the memory itself. Narrative length (i.e., word count) was, therefore, included as a covariate in subsequent analyses involving narrative variables to control for the potential influence of verbosity. This approach would reduce within-group variance, allowing more accurate estimates of the effects of independent variables, as well as ensuring that differences found between groups were not just reflecting that one group wrote more than another (Streiner, 2016).^2^

Each SDM narrative variable was analyzed with a 2 (Culture: European American vs. Chinese) × 2 (Gender: men vs. women) ANCOVA, with narrative length as a covariate^3^. Table 1 displays the estimated marginal means (adjusted for narrative length) and standard errors for the SDM narrative characteristics by culture and gender.

No significant main effect or interaction was found for meaning integration (*p*s > 0.14). Narrative length was a significant covariate, *F*(1, 235) = 8.89, *p* = 0.003, *η_p_*^2^ = 0.04, where participants who wrote longer narratives tended to report higher levels of meaning integration, 95% CI = [0.000, 0.002].

No significant main effect of culture emerged for the number of positive emotion words used, *p* = 0.35. There was a significant main effect of gender, *F*(1, 235) = 10.11, *p* = 0.002, *η_p_*^2^ = 0.04, qualified by a Gender × Culture interaction, *F*(1, 235) = 5.45, *p* = 0.02, *η_p_*^2^ = 0.02. The Bonferroni corrected post hoc test revealed that Chinese women mentioned more positive emotions than Chinese men, 95% CI = [0.35, 1.06], whereas no significant gender difference emerged among European Americans, 95% CI = [−0.25, 0.46]. Narrative length was a significant covariate, *F*(1, 235) = 52.95, *p* < 0.001, *η_p_*^2^ = 0.18, with longer narratives containing more positive emotion words than shorter narratives, 95% CI = [0.002, 0.003].

No significant main effect of culture was found for the number of negative emotion words used, *p* = 0.82. There was a significant main effect of gender, *F*(1, 235) = 32.15, *p* < 0.001, *η_p_*^2^ = 0.12, qualified by a Gender × Culture interaction, *F*(1, 235) = 12.82, *p* < 0.001, *η_p_*^2^ = 0.05. Chinese women used more negative emotion words than did Chinese men, 95% CI = [1.34, 2.50], whereas a gender difference did not emerge among European Americans, 95% CI = [−0.15, 1.01]. Narrative length was a significant covariate, *F*(1, 235) = 101.86, *p* < 0.001, *η_p_*^2^ = 0.30, where longer narratives contained more negative emotion words than shorter narratives, 95% CI = [0.005, 0.007].

In terms of specificity, the main effect of culture was significant, *F*(1, 235) = 17.28, *p* < 0.001, *η_p_*^2^ = 0.07. European Americans recalled significantly more specific memories than did Chinese participants, 95% CI = [0.26, 0.72]. No significant gender difference (*p* = 0.26) or Gender × Culture interaction (*p* = 0.43) was found. Narrative length was a significant covariate, *F*(1, 235) = 4.49, *p* = 0.04, *η_p_*^2^ = 0.02, where participants who wrote longer narratives demonstrated greater memory specificity than participants who wrote shorter narratives, 95% CI = [0.000, 0.001].

For event type, the main effect of culture on life-threatening-event content was significant, *F*(1, 235) = 4.12, *p* = 0.04, *η_p_*^2^ = 0.02, where European Americans recalled more memories focusing on life-threatening events than did Chinese participants. No significant main effect of gender (*p* = 0.19) or Gender × Culture interaction (*p* = 0.84) emerged. For relationship-focused content, the main effects of culture and gender and their interaction were not significant (*ps* > 0.23). Similarly, for achievement-focused content, the main effects of culture and gender and their interaction were not significant (*ps* > 0.27).

Regarding the other/self ratio, the main effect of culture was significant, *F*(1, 235) = 16.79, *p* < 0.001, *η_p_*^2^ = 0.07, where Chinese participants made more mentions of others relative to themselves than did European Americans, 95% CI = [0.16, 0.45]. No significant gender difference (*p* = 0.19) or Gender × Culture interaction (*p* = 0.68) was found.

### 3.2. Memory Phenomenological Qualities

Participants’ ages at the time of the memory events were similar across cultures (Euro-American: *M* = 15.18 years, *SD* = 3.54; Chinese: *M* = 14.51, *SD* = 3.40) and genders (men: *M* = 14.77 years, *SD* = 3.75; women: *M* = 14.88 years, *SD* = 3.34). Age of events was therefore not considered further. Participants’ ratings on emotional intensity were averaged across the four positive emotions (happiness, surprise, interest, and pride) and eight negative emotions (sadness, anger, fear, shame, disgust, guilt, embarrassment, contempt), respectively, across the two memories, which were then submitted to analysis. Table 2 presents the means and standard deviations for memory ratings by culture and gender.

A 2 (Culture: European American vs. Chinese) × 2 (Gender: men vs. women) ANOVA was conducted on each memory rating. There was a main effect of culture on positive emotional intensity, *F*(1, 236) = 16.16, *p* < 0.001, *η_p_*^2^ *=* 0.06, where European Americans rated positive emotions as more intense than did Chinese participants, 95% CI = [0.37, 1.07]. There was no significant main effect of gender (*p* = 0.06) or Gender × Culture interaction (*p* = 0.15) for positive emotion intensity. For negative emotion intensity, there were no significant main effects of culture (*p* = 0.78), gender (*p* = 0.08), or their interaction (*p* = 0.76).

For memory clarity ratings, neither culture (*p* = 0.96) nor gender (*p* = 0.09) showed a significant main effect, but the Gender × Culture interaction was significant, *F*(1, 236) = 5.58, *p* = 0.02, *η_p_*^2^ = 0.02. While European American women reported higher clarity than did Chinese women, *p* = 0.04, 95% CI = [0.01, 0.57], the cultural difference was not significant among men, *p* = 0.16, 95% CI = [−0.66, 0.11]. Alternatively, while European American women rated their memories as clearer than European American men, *p* = 0.004, 95% CI = [−0.83, −0.16], no significant gender difference emerged among Chinese. Finally, no significant main effect of culture (*p* = 0.88), gender (*p* > 0.99) or Gender × Culture interaction (*p* = 0.90) was found for personal importance ratings.

### 3.3. Relations of SDMs to Well-Being

Descriptive data of well-being measures, including the Flourishing Scale (FS), the Depression Scale, and SPANE-positive and SPANE-negative affect states are shown in Table 3. To obtain a comprehensive index of psychological well-being, standardized scores (z-scores) were computed for each measure, and a composite well-being score was calculated using the following formula, as adapted from Q. Wang and Suo (2023). High scores indicate greater positive functioning and positive affect, coupled with low depressive symptoms and negative affect.

Composite Well-being = ${(Flourishing}_{std}+{SPANE_{positive}}_{std})-(SPAN{E_{negative}}_{std}+{ADS}_{std}$).

We conducted a 2 (Culture: European American vs. Chinese) × 2 (Gender: men vs. women) ANOVA on each well-being measure. There was a main effect of culture on flourishing, *F*(1, 236) = 14.58, *p* < 0.001, *η_p_*^2^ = 0.06, where European American participants scored higher than Chinese participants. No significant main effect of gender (*p* = 0.51) or Gender × Culture interaction (*p* = 0.91) was found for flourishing. There was also a main effect of culture on depression, *F*(1, 236) = 14.90, *p* < 0.001, *η_p_*^2^ = 0.06, where European Americans scored higher than Chinese participants. No significant main effect of gender (*p* = 0.46) or Gender × Culture interaction (*p* = 0.69) was found for depression. No significant main effect or interaction was found for SPANE-positive (*p*s > 0.22), SPANE-negative (*p*s > 0.07), or the composite well-being (*p*s > 0.85).

We then examined the relations of the SDM narrative and phenomenological quality variables to well-being in each culture group. Table 4 presents the correlations between SDM characteristics and psychological well-being by culture. Meaning integration was positively associated with flourishing (*r* = 0.20) and composite well-being (*r* = 0.25) and negatively associated with depression (*r* = −0.21) and SPANE-negative affect states (*r* = −0.27) among European Americans, with only the association with SPANE-negative affect states being stronger for European Americans than Chinese participants. Emotional expressivity was also related to well-being. Positive emotion word use was correlated with higher SPANE-positive affect states in both groups (*r*s = 0.20, 0.21) and lower SPANE-negative affect states among European Americans (*r* = −0.18), whereas negative emotion word use was associated with more SPANE-negative affect states among Chinese (*r* = 0.19).

Content themes also showed culturally distinct patterns. Among European Americans, achievement-related memories were associated with lower depression (*r* = −0.18), and life-threatening-event memories were associated with higher SPANE-negative affect states (*r* = 0.19). Among Chinese participants, greater memory specificity was correlated with lower depression (*r* = −0.20).

Positive emotional intensity ratings were linked to higher flourishing (*r*s = 0.24, 0.34) and composite well-being (*r*s = 0.21, 0.33) across cultures. Positive emotional intensity was also associated with higher SPANE-positive affect states among Chinese (*r* = 0.38) and showed similarly small negative correlations with depression in European Americans (*r* = −0.19, *p* < 0.05) and Chinese participants (*r* = −0.18, *ns*), with no evidence that the association differed by culture. Negative emotional intensity ratings showed the opposite pattern, relating to greater depression (*r*s = 0.38, 0.20), higher SPANE-negative affect states (*r*s = 0.28, 0.31), and lower flourishing (*r*s = −0.20, −0.18) and composite well-being (*r*s = −0.28, −0.26) in both groups. Finally, clarity (*r* = 0.21) and personal importance ratings (*r* = 0.23) were respectively related to higher SPANE-positive affect states among European Americans and Chinese participants.

It should be noted that these bivariate correlations are presented descriptively and were used to guide the selection of predictors for the regression models. Given the large number of correlations reported, we applied a Benjamini–Hochberg false-discovery-rate (FDR) correction as a sensitivity analysis. Under FDR control (*p_adjusted_* < 0.05), the associations that remained significant were concentrated on emotional intensity ratings (i.e., positive intensity with flourishing, SPANE-positive, and composite well-being in the Chinese sample, and negative intensity with depression in the European American sample, and with composite well-being and SPANE-negative in both samples) and meaning integration with lower SPANE-negative among European Americans.

Next, we ran a series of multiple regression analyses to test the relations of SDM variables to well-being (McAdams & Guo, 2015; Q. Wang & Suo, 2023). In each regression model, culture (1 = China, 0 = U.S.), gender (1 = men, 0 = women), and one SDM variable that was significantly correlated with a well-being measure in at least one cultural group were entered as predictors of that well-being measure. Narrative length was included as a covariate when a narrative variable was a predictor. To test whether culture moderated the relation between memory and well-being, we also entered a two-way interaction between culture and the memory variable in each model. Table 5 shows the results of the multiple regression analyses.

#### 3.3.1. Flourishing

Meaning integration, *t*(234) = 2.03, *p* = 0.04, 95% CI = [0.06, 3.69], and higher positive emotional intensity ratings predicted higher flourishing, *t*(235) = 2.47, *p* = 0.01, 95% CI = [0.26, 2.31], while higher negative emotional intensity ratings predicted lower flourishing, *t*(235) = −2.09, *p* = 0.04, 95% CI = [−2.51, −0.07].

Culture remained a significant predictor in the models with meaning integration and positive emotional intensity ratings (*ps* = 0.02), where European Americans reported higher flourishing than Chinese participants, but not in the model with negative emotional intensity ratings (*p* = 0.07). None of the memory variables had a significant interaction with culture (*ps* > 0.17).

#### 3.3.2. Depression

Meaning integration, *t*(234) = −2.55, *p* = 0.01, 95% CI = [−6.12, −0.78], positive emotion words, *t*(234) = −1.99, *p* = 0.05, 95% CI = [−6.54, −0.03], and achievement content predicted lower depression, *t*(234) = −2.19, *p* = 0.03, 95% CI = [−5.91, −0.31]. Furthermore, while higher positive emotional intensity ratings predicted lower depression, *t*(235) = −2.29, *p* = 0.02, 95% CI = [−3.39, −0.25], higher negative emotional intensity ratings predicted higher depression, *t*(235) = 4.63, *p* < 0.001, 95% CI = [2.36, 5.86].

Culture remained a significant predictor in the models with meaning integration, positive emotion words, achievement event content, and positive emotional intensity ratings (*ps* < 0.01), where European Americans reported higher depression than did Chinese, but not in the model with specificity or negative emotional intensity ratings (*ps* > 0.22). None of the memory variables had a significant interaction with culture (*ps* > 0.12).

#### 3.3.3. SPANE-Positive Affect States

The greater use of positive emotion words predicted greater SPANE-positive affect states, *t*(234) = 2.19, *p* = 0.03, 95% CI = [0.13, 2.47]. While culture was not a significant predictor in any model (*ps* > 0.08), a significant Culture × Positive emotional intensity interaction emerged, *B* = 0.97, *SE* = 0.40, *t*(235) = 2.43, *p* = 0.02, 95% CI = [0.19, 1.75]. Higher positive emotional intensity ratings significantly predicted higher SPANE-positive affect states among Chinese participants, *B* = 1.38, *SE* = 0.29, *t*(119) = 4.77, *p* < 0.001, 95% CI = [0.81, 1.96], but not among European Americans (*p* = 0.16). In addition, gender was a significant predictor in this model, where women reported higher SPANE-positive affect states than men, *B* = −1.13, *SE* = 0.56, *t*(235) = −2.03, *p* = 0.04, 95% CI = [−2.22, −0.03].

#### 3.3.4. SPANE-Negative Affect States

Meaning integration, *t*(234) = −2.90, *p* = 0.004, 95% CI = [−2.39, −0.46], and positive emotion words, *t*(234) = −2.24, *p* = 0.03, 95% CI = [−2.53, −0.16], predicted lower SPANE-negative affect states, whereas greater negative emotional intensity ratings predicted greater SPANE-negative affect states, *t*(235) = 2.95, *p* = 0.003, 95% CI = [0.32, 1.60]. While culture was not a significant predictor in any model (*p* > 0.28), the Culture × Meaning interaction was significant, *B* = 1.46, *SE* = 0.66, *t*(234) = 2.21, *p* = 0.03, 95% CI = [0.16, 2.76]. Meaning integration significantly predicted lower SPANE-negative affect states among European Americans, *B* = −1.44, *SE* = 0.52, *t*(114) = −2.78, *p* = 0.01, 95% CI = [ −2.46, −0.41], but not among Chinese participants (*p* = 0.94).

#### 3.3.5. Composite Well-Being

Meaning integration, *t*(234) = 2.77, *p* = 0.01, 95% CI = [0.30, 1.81], and higher positive emotional intensity ratings predicted higher composite well-being score, *t*(235) = 2.35, *p* = 0.02, 95% CI = [0.08, 0.95], whereas higher negative emotional intensity ratings predicted lower composite well-being score, *t*(235) = −3.18, *p* = 0.002, 95% CI = [−1.31, −0.31]. No significant culture effect (*ps* > 0.29) or Culture × Memory interaction was found (*ps* > 0.11).

## 4. Discussion

The present study examined the construction of self-defining memories as they relate to psychological well-being in different cultural contexts. Specifically, we investigated the characteristics of SDMs reported by European American and Chinese emerging adults and their associations with psychological well-being. Our findings showed that European American and Chinese participants differed in SDM characteristics and in their links to psychological well-being. Below, we discuss our findings regarding memory characteristics, memory ratings, and their implications for psychological well-being.

### 4.1. Self-Defining Memory Narrative Characteristics Across Cultures

Consistent with our prediction, European Americans provided more specific SDM narratives than Chinese participants. This pattern aligns with prior findings that individuals from Western cultures are more likely to recall one-time, specific events than those from Eastern cultures (Q. Wang, 2001, 2009; Q. Wang et al., 2011). Specific SDMs may facilitate an independent sense of the self, in line with the European American cultural emphasis on individuality and uniqueness (Ross & Wang, 2010). Furthermore, as hypothesized, Chinese participants provided more socially oriented narratives, mentioning others more frequently relative to the self than did European Americans. This finding is consistent with prior research reporting a greater social focus among East Asian autobiographical narratives (Q. Wang, 2001; Q. Wang & Ross, 2005; Q. Wang & Conway, 2004), in line with the interdependent self-construal and the cultural emphasis on relational concerns and social embeddedness in East Asian contexts (Markus & Kitayama, 1991).

Contrary to our expectation, meaning integration did not differ between the two cultural groups. It is possible that deriving meaning from personally important experiences is a relatively common process across cultures (Reese et al., 2017; Q. Wang, 2013, 2021). Notably, our coding only captured the presence of meaning integration rather than the type of meaning that was derived from past experiences. Prior work suggests that meaning-making in East Asian contexts often involves generating moral lessons and broader life principles, a form that has been linked to Confucian values (Zhang et al., 2005), whereas meaning-making in European American contexts often emphasizes silver linings and reappraisals to render negative events more emotionally positive (McAdams, 2006b; Q. Wang & Conway, 2004; Q. Wang, 2013). Clearly, more work is called for to understand aspects of narrative meaning-making in the cultural context.

In addition, Chinese and European American participants showed similar tendencies to remember achievement- and relationship-related SDMs. These cultural similarities might reflect the characteristics of the college student samples, young people for whom academic achievement and relationships were both important at this life stage. Prior research has also found that the social-bonding functions of autobiographical memory were similarly endorsed in both cultural contexts (Y. Wang & Singer, 2021). On the other hand, European Americans recalled more life-threatening events (LTEs) than Chinese participants, such as severe illness, death, or accidents involving themselves or close others, a finding consistent with prior work showing that Americans more often report traumatic or high-impact experiences as self-defining than Chinese people (Jiang et al., 2023).

Interestingly, gender differences in the emotional expressivity of SDMs emerged only among Chinese participants, with Chinese women expressing both more positive and negative emotions than Chinese men, independent of narrative length. Prior work with Western samples has also observed that women tend to use more emotional words than men in their narratives (Bohanek & Fivush, 2010; Grysman et al., 2016), although gender differences in narrative emotional expressivity are not always present and seem to be context-dependent (Chaplin & Aldao, 2013; Davis et al., 2012; Grysman & Wang, 2021; Pennebaker & Graybeal, 2001). Given that the current gender findings were only as predicted in one cultural group but not the other, further corroboration is required to afford any reliable explanations.

### 4.2. Memory Phenomenological Qualities Across Cultures

In terms of memory ratings, European Americans reported higher positive emotional intensity for their SDMs than did Chinese participants, while the two groups did not differ on negative emotional intensity. This difference may reflect different emotion beliefs and display norms across cultures. In East Asian cultures like China, emotional moderation is often valued as a means of preserving interpersonal harmony, and individuals tend not to directly express strong emotions, including positive ones (Yang & Wang, 2019). In contrast, American cultural norms often emphasize maximizing positive emotions to enhance the self (Ma et al., 2018). Prior work has similarly documented higher positive emotional intensity in U.S. than Chinese samples (Davis et al., 2012).

Regarding memory clarity, European American women reported higher clarity than did European American men, whereas no such gender difference was observed among Chinese participants. One possible explanation is that European American women may engage more frequently in reflective elaboration of personally meaningful events, as previous research has observed that women in Western contexts tend to rehearse, discuss, and process autobiographical memories more frequently than men in Western contexts (Grysman & Hudson, 2013; Harbus, 2011; Q. Wang, 2013), which may potentially contribute to the greater clarity of women’s memories. In contrast, gender differences in cognitive or emotional elaboration may be less pronounced in China, where cultural norms emphasize moderation and less frequent use of memory to understand the self (Liao et al., 2015; Maki et al., 2014; Ross & Wang, 2010). Finally, participants from the two groups rated their SDMs as similarly important, which seems reasonable given the conceptualization of SDMs as inherently self-relevant, personally meaningful, and emotionally intense (Y. Wang & Singer, 2021; Jiang et al., 2023).

### 4.3. Relating Self-Defining Memories to Well-Being

European Americans reported higher depression and flourishing experience than did Chinese, although there were no significant differences in the composite well-being score. The co-occurrence of elevated positive and negative well-being indicators among European Americans suggests that positive and negative affective experiences may be evaluated more independently in Western contexts, given the analytical thinking, whereas the holistic and dialectical views of emotions in Chinese culture may contribute to more balanced reports across well-being domains (De Vaus et al., 2018; Yang & Wang, 2019).

Partially consistent with our prediction, meaning integration was associated with lower depression and higher flourishing and overall well-being among European Americans, whereas these patterns did not emerge among Chinese participants, although we did not find evidence that culture significantly moderated these associations. These findings are in line with prior work linking meaning integration with emotional regulation and psychological adjustment, particularly in the Western cultural context (Cox & McAdams, 2014; Gillies & Neimeyer, 2006; McAdams et al., 2001; Park, 2010). Notably, culture moderated the relation between meaning integration and negative affect states, such that meaning integration predicted reduced negative affect states only among European Americans and not Chinese participants. This lack of association in the Chinese group is consistent with previous work showing that meaning integration can co-occur with reports of academic stress among Chinese college students (Jiang et al., 2023). One possible explanation, based on the literature, is that meaning integration may be more closely tied to reappraisals for reducing negative affect in the European American than Chinese context (Duman & Wang, 2025; Spencer-Rodgers et al., 2010; Q. Wang & Conway, 2004).

Consistent with our expectations, emotional expressions in SDM narratives were related to well-being among both European American and Chinese participants. Positive emotional word use was correlated with higher positive affect states in both cultural groups, but with lower negative affect states only among European Americans. Regression analyses further showed that the greater use of positive emotional words was associated with higher positive affect states, lower negative affect states, and lower depression, regardless of culture. These patterns are in line with the theoretical notion that emotional expressiveness in SDMs may reflect broader affective tendencies, whereby rich positive emotional expressions in narratives are associated with an overall positive affect state and well-being (Blagov et al., 2022; Lilgendahl & McAdams, 2011; Nourkova & Vasilenko, 2017; J. A. Singer & Blagov, 2004). These findings also highlight the potential utility of positive emotional word use as a linguistic marker of well-being in the context of self-defining memories. In addition, negative emotional word use was correlated with higher SPANE-negative affect states among Chinese participants, but not European Americans. This association did not replicate in the regression analysis and should be interpreted with caution.

Furthermore, in line with our expectations, perceived positive and negative emotional intensity of SDMs showed largely consistent associations with psychological well-being across cultures. Higher positive emotional intensity was related to greater flourishing and overall well-being in both cultural groups, while its relation to lower depression reached significance only among European Americans, although we did not find evidence for moderation by culture. Furthermore, higher negative emotional intensity was related to lower flourishing and overall well-being and to higher depression and negative affect states in both groups. These findings are consistent with the broader evidence linking positive emotional experiences with life satisfaction and linking intense negative emotional experiences with distress and depressive symptoms (Lyubomirsky et al., 2005; Tappolet & Rossi, 2015; Çili & Stopa, 2015; Le Nguyen & Fredrickson, 2017). Notably, the link between positive emotional intensity of SDMs and positive affect states was significant only among Chinese participants, but not among European Americans. Previous research suggests that a greater match between individual affect and cultural norms of emotion expression is associated with better psychological well-being (Kim & Mesquita, 2025; Yang & Wang, 2019), although high-intensity positive emotions tend to be less valued in East Asian than Western cultures (Tsai et al., 2006). Future research is needed to clarify why, in the context of SDMs, positive emotion intensity was more strongly related to well-being among Chinese than European American participants.

In addition, consistent with our prediction, achievement content was related to lower depression among European Americans, but not Chinese participants, although culture was not found to be a significant moderator. Prior work similarly links academic achievement experiences to subjective well-being among students (Bücker et al., 2018). Notably, although personal achievement is in line with the culturally valued self-goals of autonomy and competence in the European American context (Q. Wang et al., 2024), it is also regarded as of paramount importance for learning and social purposes in Chinese culture (Li & Wang, 2004; Wei et al., 2025). Achievement themes may thus be relevant to psychological adjustment across cultures, particularly in student samples where achievements are often central to identity and self-evaluation. In terms of LTE content, although it was correlated with higher negative affect states among European Americans but not Chinese participants, the association did not hold in the regression analysis.

Interestingly, positive affect states were associated with the personal importance of SDMs for Chinese participants and with memory clarity for European Americans. Although these associations did not hold in the regression analyses, they suggest that the same well-being measure can be associated with different phenomenological features of SDMs across cultures. Finally, contrary to our prediction, narrative specificity was not associated with well-being. Prior research has generally linked reduced memory specificity to affective disorders (Blagov & Singer, 2004; Moore et al., 1988; J. A. Singer & Salovey, 1993; J. M. G. Williams et al., 2007; J. M. Williams & Broadbent, 1986; J. M. G. Williams & Dritschel, 1988; J. M. G. Williams & Scott, 1988). Although greater specificity was correlated with lower depression among Chinese participants, this association did not hold in the regression analysis, nor was it observed among European Americans. Future corroboration is needed to fully understand these findings.

### 4.4. Limitations and Future Directions

One limitation of the present study lies in the homogeneity of the sample, which consisted of undergraduate students from elite universities in the U.S. and China. Participants within each group likely shared similar socioeconomic backgrounds, educational experiences, and developmental contexts, and neither group could represent the diverse populations in their respective countries. These shared characteristics may limit the generalizability of the findings. Future research should aim to recruit more demographically diverse samples, such as individuals from different educational settings, age groups, and socioeconomic statuses, to determine whether the observed patterns extend to broader populations.

Furthermore, while the present study examined the construction of self-defining memories as a cultural process at the group level and further linked the memories to psychological well-being at the individual level, the findings can be enriched by examining the impact of culture at other levels to address additional research questions (Q. Wang, 2018, 2026). For example, cohort and longitudinal studies can help to reveal the impact of changing cultural values and practices on self-defining memories in relation to psychological functioning (Chen, 2018; Greenfield, 2018). Studies examining individual cultural attitudes or identification can help to reveal personal agency in the cultural construction of memory and well-being (Guan & Wang, 2022; Q. Wang, 2001). Studies may also examine the manifestation of culture during social interactions, such as memory conversations (Koh & Wang, 2021), and in specific situations where a particular cultural framework becomes salient (Q. Wang & Ross, 2005).

We recognize that our study utilized a correlational design, which limits the conclusions that can be drawn about directionality in the associations between SDM characteristics and well-being. It is likely that the relations are bidirectional. Longitudinal and experimental studies will help to further reveal these directional dynamics. In addition, our well-being measures indexed affect states (i.e., positive and negative affect), subjective functioning (i.e., flourishing), and distress (i.e., depressive symptoms). The findings cannot be automatically generalized to other facets of well-being (e.g., social well-being; Helliwell & Putnam, 2004) or objective indicators of functioning (e.g., physical health; Voukelatou et al., 2021). Future research should examine the construction of SDMs as healthy coping mechanisms for additional well-being outcomes.

In addition, the current sample size might not have provided sufficient power to detect small effects. Post hoc sensitivity analyses indicated that the study was powered to detect effects in the small-to-moderate range (i.e., approximately *f* ≈ 0.18; *η_p_*^2^ ≈ 0.03; *d* ≈ 0.36 with ~80% power), whereas smaller effects would be less reliably detected. Thus, nonsignificant effects, particularly ones near the small-effect range, should be interpreted with caution, and the absence of statistical significance should not be taken as evidence of no effect. Also, given the volume of testing, particularly in the correlation matrix and multiple regressions, we wish to highlight findings that are hypothesized a priori and robust across specifications and sensitivity checks, while interpreting smaller, isolated effects as exploratory. Future cross-cultural research replicating the present findings will be important for clarifying these patterns.

Finally, each participant provided two SDMs, which were averaged and submitted to analyses. Although these narratives provide valuable insights into self-relevant experiences in different cultural contexts, participants may have experienced many self-defining memories that varied in function, emotionality, and thematic content. Relying on only two narratives likely limited the representativeness of SDM characteristics, but this study was merely an initial step in mapping out the different emotional processes experienced in one aspect of autobiographical memory across two cultures. Future research should collect a broader range of SDMs (Blagov et al., 2022) and use multi-level modeling approaches to test the relationships of each memory to well-being to better capture within-person variability.

## 5. Conclusions

The present study yielded evidence that self-defining memory characteristics and their associations with psychological well-being might vary in different cultural contexts. European Americans produced more specific SDMs, whereas Chinese participants provided more socially oriented SDMs. Gender differences also emerged among Chinese participants, but not European Americans, with women expressing more positive and negative emotions in their narratives than men. In terms of well-being, European Americans reported both higher depressive symptoms and higher flourishing than Chinese participants. While several SDM characteristics were associated with well-being in both groups, some associations differed across the two cultural contexts. Positive emotional intensity was associated with positive affect states only among Chinese participants, whereas meaning integration was associated with lower negative affect states only among European Americans. Together, these findings suggest that self-defining memories are meaningfully related to well-being, a constructive process that can be shaped by culture.

## Figures and Tables

**Table 1:** Descriptive statistics of SDM dimensions by culture and gender.

	Chinese				Euro-American			
	Women		Men		Women		Men	
Memory Characteristics	*M*	*SE*	*M*	*SE*	*M*	*SE*	*M*	*SE*
Meaning integration	0.79	0.10	0.63	0.14	0.62	0.10	0.79	0.13
Positive emotion words	0.88	0.11	0.18	0.16	0.72	0.11	0.62	0.15
Negative emotion words	2.34	0.18	0.42	0.25	1.65	0.18	1.22	0.25
Specificity	1.05	0.09	1.24	0.12	1.61	0.09	1.65	0.12
Relationship content	0.61	0.08	0.60	0.11	0.39	0.08	0.60	0.11
LTE content	0.19	0.06	0.12	0.08	0.36	0.06	0.26	0.08
Achievement content	0.73	0.09	0.89	0.12	0.89	0.09	0.83	0.12
Other/self ratio	0.77	0.05	0.72	0.08	0.50	0.06	0.39	0.07

**Table 2:** Descriptive statistics of memory ratings by culture and gender.

	Chinese				European American			
	Women		Men		Women		Men	
Memory Ratings	*M*	*SD*	*M*	*SD*	*M*	*SD*	*M*	*SD*
Positive emotion intensity	2.04	1.19	2.63	1.44	3.01	1.30	3.08	1.41
Negative emotion intensity	1.71	0.96	1.50	0.94	1.72	1.20	1.42	1.08
Clarity	4.63	0.82	4.71	1.01	4.92	0.83	4.43	0.99
Personal importance	4.85	0.89	4.87	0.92	4.85	0.88	4.83	0.77

**Table 3:** Descriptive statistics of well-being measures by culture and gender.

	Chinese				European American			
	Women		Men		Women		Men	
Well-Being Measures	*M*	*SD*	*M*	*SD*	*M*	*SD*	*M*	*SD*
Flourishing	41.53	7.73	40.71	9.85	45.51	5.85	44.93	9.31
Depression	35.21	10.13	34.68	11.25	41.97	12.92	40.17	12.55
SPANE-positive	21.26	4.38	20.76	4.48	21.61	3.67	20.69	4.68
SPANE-negative	16.41	4.75	15.61	3.38	16.29	3.99	15.00	4.44
Composite well-being	−0.01	3.17	0.01	3.37	−0.05	3.05	0.09	3.90

**Table 4:** Associations between memory characteristics and well-being measures by culture.

	Chinese					European American					Z-scores				
Memory Characteristics	FS	DP	SPANE-P	SPANE-N	C-WB	FS	DP	SPANE-P	SPANE-N	C-WB	FS	DP	SPANE-P	SPANE-N	C-WB
Meaning integration	0.05	−0.05	0.09	0.02	0.05	0.20 *	−0.21 *	0.14	−0.27 *	0.25 *	−1.17	1.25	−0.39	2.27 *	−1.57
Positive emotion words	0.05	−0.01	0.21 *	0.00	0.08	0.03	−0.15	0.20 *	−0.18 *	0.17	0.15	1.08	0.08	1.40	−0.70
Negative emotion words	−0.07	0.09	−0.09	0.19 *	−0.14	−0.01	0.12	0.06	0.08	−0.04	−0.46	−0.23	−1.15	0.86	−0.77
Specificity	0.13	−0.20 *	−0.05	−0.15	0.13	0.03	−0.00	0.01	0.08	−0.01	0.77	−1.55	−0.46	−1.77	1.08
LTE content	0.14	−0.04	−0.02	−0.05	0.07	−0.05	0.15	−0.09	0.19 *	−0.14	1.46	−1.46	0.54	−1.85	1.61
Achievement content	−0.08	−0.03	−0.01	0.02	−0.02	0.17	−0.18 *	0.06	−0.17	0.17	−1.93	1.16	−0.54	1.47	−1.47
Positive emotion intensity	0.34 **	−0.18	0.38 **	−0.17	0.33 **	0.24 *	−0.19 *	0.13	−0.14	0.21 *	0.84	0.08	2.06 *	−0.24	0.99
Negative emotion intensity	−0.18 *	0.20 *	−0.13	0.31 **	−0.26 *	−0.20 *	0.38 **	−0.08	0.28 *	−0.28 *	0.16	−1.51	−0.39	0.25	0.16
Importance	0.17	0.06	0.23 *	0.15	0.06	0.09	−0.03	0.10	−0.03	0.08	0.62	0.69	1.02	1.39	−0.15
Clarity	0.12	0.14	0.07	0.06	−0.004	0.06	−0.09	0.21 *	−0.05	0.12	0.46	1.77	−1.09	0.84	−0.92

*Note.* FS = Flourishing Scale, DP = Depression, SPANE-P = SPANE-Positive, SPANE-N = SPANE-Negative, and C-WB = Composite well-being score. Z-scores were calculated using the Fisher r-to-z transformation comparing the strength of associations across Chinese and European American samples. Bolded entries reflect significant associations after BH-FDR correction. * *p* < 0.05. ** *p* < 0.01.

**Table 5:** Multiple regression results (b) for SDM characteristics predicting psychological well-being.

	FS	DP	SPANE-P	SPANE-N	C-WB
Meaning integration	1.88 *	−3.45 *	-	−1.43 **	1.06 *
Positive emotion words	-	−3.29 *	1.30 *	−1.34 *	-
Negative emotion words	-	-	-	0.28	-
Specificity	-	−0.05	-	-	-
LTE content	-	-	-	1.42	-
Achievement content	-	−3.11 *	-	-	0.77
Positive-emotion intensity ratings	1.29 *	−1.82 *	0.40	-	0.52 *
Negative-emotion intensity ratings	−1.29 *	4.11 ***	-	0.96 **	−0.81 **
Importance ratings	-	-	0.49	-	-
Clarity ratings	-	-	0.85	-	-

*Note.* In each regression analysis, one SDM variable was entered along with culture, gender, narrative length (for narrative variable only), and a 2-way interaction between culture and the SDM variable as predictors of one well-being measure that was significantly correlated with the SDM variable in at least one cultural group. FS = Flourishing Scale, DP = depression, SPANE-P = SPANE-positive, SPANE-N = SPANE-negative, and C-WB = composite well-being score. * *p* < 0.05. ** *p* < 0.01. *** *p* < 0.001.

## Data Availability

The data presented in this study are openly available in Open Science Forum [OSF: https://osf.io/65q2k], accessed on 8 March 2026.

## Notes

1 We evaluated the measurement structure of the emotion ratings using confirmatory factor analysis. A two-factor model (positive vs. negative affect) provided good fit and reproduced the intended item groupings (see Supplementary Materials). Accordingly, we used mean composites for positive and negative emotional intensity in primary analyses. Factor-score-based analyses yielded substantively identical conclusions.

2 Additional analyses were conducted without including narrative length as a covariate. The results were largely consistent with those reported in the main text (see Supplementary Materials).

3 To address potential Type I error when dependent variables within a domain were correlated, we conducted multivariate sensitivity analyses for each set of related outcomes (i.e., narrative characteristics, memory ratings, and well-being). Results were consistent with the univariate analyses (See Supplementary Materials).

## Supplementary Materials

The following supporting information can be downloaded at: https://www.mdpi.com/article/10.3390/bs16030408/s1, Table S1: Correlations between CFA-derived emotion factor scores and well-being indicators by culture. References (Cole et al., 2007; Kline, 2023) are cited in the Supplementary Materials.
