# Supplementary material for: Self-Defining Memories and Well-Being in European American and Chinese Emerging Adults
Source: Behav Sci (Basel). 2026 Mar 11;16(3):408. doi: 10.3390/bs16030408 (PMC13023813; doi:10.3390/bs16030408)
Supplement: Supplementary file 1 [file behavsci-16-00408-s001.zip › behavsci-4063536-supplementary.pdf]

Supplementary Materials

Confirmatory Factor Analyses for the 12 Memory Emotional Intensity Ratings

To evaluate whether the a priori positive (happy, interested, surprised, proud) and negative (sad, angry, fearful, ashamed, disgusted, guilty, embarrassed, contemptful) emotion composites correspond to the latent structure of the 12 emotion ratings, we conducted confirmatory factor analyses using MLMV estimation with standardized solutions ($N = 240$).

We first estimated a bifactor model consisting of a general "Emotion" factor loading on all 12 items and two group factors (Positive and Negative) loading onto their respective indicators. This model demonstrated convergence issues, and the fitted solution was not full rank, indicating identification problems. Consistent with SEM best practices, the bifactor solution was treated as unreliable and not interpreted (see Kline, 2023).

We then estimated a two-factor model with correlated Positive and Negative latent factors and allowed residuals between conceptually overlapping items (i.e., ashamed–embarrassed, ashamed–guilty, angry–guilty, angry–contemptful) to covary (Cole et al., 2007).

The two-factor model converged successfully and showed mixed-to-adequate fit by incremental indices but elevated RMSE and slightly low TLI: $\chi^2(49) = 178.98$, $p < .001$; CFI = .913; TLI = .883; RMSEA = .105, 90% CI [.089, .122]; SRMR = .079. All items loaded in the expected direction on their designated factors, although, surprised (Positive) and contemptful (Negative) showed notably weak standardized loadings. The Positive and Negative factors were strongly negatively correlated ($r = -.856$, $p < .001$). The four specified correlated residuals among negative emotions were all statistically significant (ps < .001).

Overall, the converged CFA results were largely consistent with the intended positive vs. negative organization of the emotion adjectives, supporting the use of the Positive and Negative

emotion composites in the main analyses. At the same time, model fit was mixed (e.g., RMSEA = .105; TLI = .883), and two items showed comparatively weak loadings, suggesting that these specific indicators may contribute less strongly to their respective latent dimensions in this dataset.

To evaluate whether associations between memory-related emotional intensity and well-being were sensitive to how emotional intensity was scored, we repeated the correlational analyses using CFA-derived factor scores for Positive and Negative Emotional Intensity. As shown in Supplemental Table S1, the factor-score correlations with well-being indicators largely mirrored the pattern obtained using composite scores. A small number of associations differed in statistical significance, particularly with Flourishing in the European American sample and with SPANE-Positive and SPANE-Negative in Chinese sample.

Supplemental Table S1.

Correlations between CFA-derived emotion factor scores and well-being indicators by culture

	Chinese		European American	
	Positive Emotional Intensity	Negative Emotional Intensity	Positive Emotional Intensity	Negative Emotional Intensity
Composite Well-Being	.31***	-.30***	.19*	-.22*
Flourishing	.27**	-.22*	.17	-.16
SPANE-Positive	.35***	-.25**	.08	-.06
SPANE-Negative	-.22*	.29***	-.17	.22*
Depressive Symptoms	-.17	.22*	-.22*	.30***

Note. *$p < .05$. **$p < .01$. ***$p < .001$.

Multivariate Analyses of Variance on Narrative Characteristics, Memory Ratings, and Well-Being Measures

A multivariate analysis of covariance (MANCOVA) tested effects of culture, gender, and their interaction on eight narrative characteristics, controlling for narrative length.

Using Wilks' lambda, there were significant multivariate effects of culture, Wilks' Λ = .835, $F(8, 228) = 5.64$, $p < .001$, $\eta_p^2 = .165$, gender, Wilks' Λ = .815, $F(8, 228) = 6.45$, $p < .001$, $\eta_p^2 = .185$, and the Culture × Gender interaction, Wilks' Λ = .915, $F(8, 228) = 2.64$, $p = .009$, $\eta_p^2 = .085$. The multivariate effects of narrative length were also significant, Wilks' Λ = .570, $F(8, 228) = 21.53$, $p < .001$, $\eta_p^2 = .430$. Follow-up univariate tests indicated that the multivariate effect of culture was driven by differences in life-threatening event content ($F(1, 235) = 4.12$, $p = .043$, $\eta_p^2 = .017$), specificity ($F(1, 235) = 17.28$, $p < .001$, $\eta_p^2 = .069$), and other/self ratio ($F(1, 235) = 16.78$, $p < .001$, $\eta_p^2 = .067$). Gender multivariate effect emerged for positive emotion words ($F(1, 235) = 10.11$, $p = .002$, $\eta p^2 = .041$) and negative emotion words ($F(1, 235) = 32.15$, $p < .001$, $\eta_p^2 = .120$). Finally, Culture × Gender interaction was significant for positive emotion words ($F(1, 235) = 5.45$, $p = .020$, $\eta_p^2 = .023$) and negative emotion words ($F(1, 235) = 12.82$, $p < .001$, $\eta_p^2 = .052$).

Next, a MANOVA examined four memory ratings as outcomes with culture, gender and their interaction.

There was a significant multivariate effect of culture, Wilks' Λ = .902, $F(4, 233) = 6.35$, $p < .001$, $\eta_p^2 = .098$, and a significant Culture × Gender interaction, Wilks' Λ = .957, $F(4, 233) = 2.60$, $p = .037$, $\eta_p^2 = .043$. The multivariate effect of gender was not significant, Wilks' Λ = .967, $F(4, 233) = 1.98$, $p = .099$, $\eta_p^2 = .033$. Univariate tests indicated a significant culture

difference in positive emotional intensity, $F(1, 236) = 16.38$, $p < .001$, $\eta_p^2 = .065$, and the Culture × Gender interaction was significant for clarity, $F(1, 236) = 5.58$, $p = .019$, $\eta_p^2 = .023$.

Finally, a MANOVA was conducted with culture, gender, and their interaction on five well-being indicators.

There was a significant multivariate effect of culture, Wilks' $\Lambda = .605$, $F(5, 232) = 30.35$, $p < .001$, $\eta_p^2 = .395$. The multivariate effect of gender did not reach significance, Wilks' $\Lambda = .957$, $F(5, 232) = 2.09$, $p = .067$, $\eta_p^2 = .043$, and the Culture × Gender interaction was not significant, Wilks' $\Lambda = .985$, $F(5, 232) = 0.72$, $p = .612$, $\eta_p^2 = .015$. Follow-up univariate tests indicated significant culture differences for flourishing, $F(1, 236) = 14.58$, $p < .001$, $\eta_p^2 = .058$, and depressive symptoms, $F(1, 236) = 14.90$, $p < .001$, $\eta_p^2 = .059$.

Overall, the multivariate analyses of narrative characteristics, memory ratings, and well-being measures were consistent with the pattern observed in the univariate analyses reported the manuscript.

2 (Culture: European American vs. Chinese) × 2 (Gender: men vs. women) ANOVAs Without Narrative Length as a Covariate

The were significant main effects of culture, $F(1,236) = 10.39$, $p = .001$, η_p^2=.04, and gender, $F(1,236) = 7.20$, $p = .01$, η_p^2=.03, on the number of positive emotion words used. Chinese participants mentioned more positive emotions than European Americans, 95% CI = [.18, .73], and women mentioned more positive emotions than men, 95% CI = [.10, .66].

There were also significant main effects of culture, $F(1,236) = 27.00$, $p < .001$, $\eta_p^2 = .10$, and gender, $F(1,236) = 20.23$, $p < .001$, $\eta_p^2 = .08$, on the number of negative emotion words used. These main effects were qualified by a Culture × Gender interaction, $F(1,236) = 4.57$, p =.03, $\eta_p^2 = .02$. Chinese women used more negative emotion words than did Chinese men, 95% CI = [.95,

2.33], whereas gender difference did not emerge among European Americans, 95% CI = [−.11, 1.27].

The main effect of culture on specificity was significant, $F(1,236) = 12.63$, $p < .001$, $\eta_p^2 = .05$. European Americans recalled significantly more specific memories than did Chinese participants, 95% CI = [.16, .55].

The main effect of culture on other/self ratio was also significant, $F(1,236) = 26.38$, $p < .001$, $\eta_p^2 = .10$, whereby Chinese participants made more mentions of others relative to themselves than did European Americans, 95% CI = [.20, .44].

Finally, no significant main effects or Culture × Gender interaction was found on narrative content and meaning integration.

These findings are broadly consistent with the results of analyses reported in the manuscript that controlled for narrative length. Differences only emerged for life-threatening event content, where the main effect of culture observed in the covariate-adjusted model did not remain significant once narrative length was excluded, and for positive emotion words, where the Culture × Gender interaction did not emerge and the main effect of culture became significant once narrative length was excluded.

References

Cole, D. A., Ciesla, J. A., & Steiger, J. H. (2007). The insidious effects of failing to include design-driven correlated residuals in latent-variable covariance structure analysis. *Psychological Methods, 12*(4), 381–398.

Kline, R. B. (2023). *Principles and practice of structural equation modeling*. Guilford publications.
